# An alternative evolutionary pathway for the twin‐tail goldfish via *szl* gene mutation

**DOI:** 10.1002/jez.b.22811

**Published:** 2018-06-27

**Authors:** Gembu Abe, Shu‐Hua Lee, Ing‐Jia Li, Kinya G. Ota

**Affiliations:** ^1^ Laboratory of Aquatic Zoology Marine Research Station Institute of Cellular and Organismic Biology Academia Sinica Yilan Taiwan; ^2^ Laboratory of Organ Morphogenesis Department of Developmental Biology and Neurosciences Graduate School of Life Sciences Tohoku University Sendai Japan

**Keywords:** artificial selection, axial skeleton, dorsal–ventral patterning, *szl*

## Abstract

The twin‐tail of ornamental goldfish provides unique evolutionary evidence that the highly conserved midline localization of axial skeleton components can be changed by artificial selection. This morphological change is known to be caused by a nonsense mutation in one of the recently duplicated *chordin* genes, which are key players in dorsal–ventral (DV) patterning. Since all of the multiple twin‐tail ornamental goldfish strains share the same mutation, it is reasonable to presume that this mutation occurred only once in domesticated goldfish. However, zebrafish with mutated *szl* gene (another DV patterning‐related gene) also exhibit twin‐tail morphology and higher viability than *dino/chordin*‐mutant zebrafish. This observation raises the question of whether the *szl* gene mutation could also reproduce the twin‐tail morphology in goldfish. Here we show that goldfish have at least two subfunctionalized *szl* genes, designated *szlA* and *szlB*, and depletion of these genes in single‐fin goldfish was able to reproduce the bifurcated caudal fin found in twin‐tail ornamental goldfish. Interestingly, several phenotypes were observed in *szlA*‐depleted fish, while low expressivity of the twin‐tail phenotype was observed in *szlB*‐depleted goldfish. Thus, even though *szl* gene mutations may produce twin‐tail goldfish, these *szl* gene mutations might not be favorable for selection in domestic breeding. These results highlight the uniqueness and rarity of mutations that are able to cause large‐scale morphological changes, such as a bifurcated axial skeleton, with high viability and expressivity in natural and domesticated populations.

## 1 INTRODUCTION

All vertebrates exhibit conserved midline localization of the axial skeletal system (Kardong, 2012; Liem, Bemis, Walker, & Kabce, 2001). Although occasional multihead or multitail mutants have been reported across vertebrate species in natural and domesticated populations (Bateson, 1894; Korschelt, 1907; Suzuki, 2016), almost none has been established as a stable strain. In this context, the twin‐tail ornamental goldfish (*Carassius auratus*) can be considered as a unique example, since it possesses a bifurcated caudal fin and duplication of the axial skeleton within the bifurcated region (Ota & Abe, 2016; Watase, 1887). According to Chinese archives, goldfish domestication for ornamental purposes began around 1000 c.e., in the Song dynasty. After approximately 600 years of domestication, the twin‐tail goldfish appeared, and the unique caudal morphology has been maintained until present day (Chen, 1956). The mutation that is responsible for this morphology was recently discovered by molecular genetic analysis and revealed to be within the *chordin* gene (Abe et al., 2014).

Due to the integral role of the *chordin* gene in dorsal–ventral (DV) patterning, depletion of this gene has been reported to be lethal in several model organisms (Fisher & Halpern, 1999; Oelgeschläger, Kuroda, Reversade, & De Robertis, 2003; Schulte‐Merker, Lee, McMahon, & Hammerschmidt, 1997; Takashima et al., 2007). The low viability of *chordin* gene‐depleted model organisms may explain why the twin‐tail morphology is uncommon in nature (Abe et al., 2014; Ota & Abe, 2016). In fact, *chordin* gene‐mutant zebrafish (*dino/chordin*) and medaka (*chordin^UT600^*), which were derived from mutagenesis experiments, tend to exhibit severe phenotypes along with the twin‐tail morphology (Fisher & Halpern, 1999; Takashima et al., 2007). In contrast to these *chordin*‐depleted model vertebrates (Fisher & Halpern, 1999; Oelgeschläger et al., 2003; Schulte‐Merker et al., 1997; Takashima et al., 2007), the twin‐tail goldfish exhibits high survivability due to the duplication and subfunctionalized expression of *chordin* genes (*chdA* and *chdB*; Abe et al., 2014). Recent genomic and molecular sequence analyses in goldfish and common carp (*Cyprinus carpio*, the closest relative of goldfish), have suggested that a number of genes were duplicated during a lineage‐specific allotetraploidization (genome duplication with species hybridization; Abe et al., 2014; Luo et al., 2006; Luo, Standler, He, & Meyer, 2007; Xu et al., 2014). It is presumed that the two *chordin* gene paralogues are derived from this genome duplication and in twin‐tail goldfish, the function of the *chdA* gene is lost by a mutation that introduced a premature stop codon (*chdA^E127X^*; Abe et al., 2014; Luo et al., 2006, 2007; Xu et al., 2014). Since the *chdA^E127X^* allele is homozygous in all investigated ornamental twin‐tail goldfish strains, it is naturally hypothesized that this mutation occurred only once and became genetically fixed in all ornamental twin‐tail goldfish (Abe et al., 2014).

Despite the single origin of twin‐tail morphology in goldfish, it is unknown whether *chdA* is the only goldfish gene of which disruption could cause this skeletal phenotype. In fact, molecular developmental genetics experiments in zebrafish have identified several gene mutations that can produce the twin‐tail morphology. It is known that more than 18 genes (including *szl*, *tll*, and *BMPs*) cooperate with the *chordin* gene to regulate embryonic DV patterning (Langdon & Mullins, 2011; Muraoka et al., 2006; Yabe et al., 2003), and the zebrafish studies suggest that mutations in several of these DV patterning‐related genes could reproduce the twin‐tail phenotype.

Among the zebrafish mutations, it was reported that the *szl* gene mutation reproduces the twin‐tail morphology, the mutant designated as *ogon/sizzled* (Hammerschimidt et al., 1996; van Eeden et al., 1996). Interestingly, the *ogon/sizzled* mutant exhibits a higher survival rate than the *dino/chordin* zebrafish mutant (Fisher & Halpern, 1999; van Eeden et al., 1996), leading us to suspect that the twin‐tail phenotype may also be reproduced in goldfish by mutations in the *szl* gene (the hypothetical *szl* mutant is called the “*szl* type” twin‐tail goldfish). However, no *szl*‐type twin‐tail goldfish has been found in nature or domesticated populations thus far. This mismatch of expectation and reality begs a simple question: Is there any biological reason why the *szl*‐type twin‐tail goldfish has not been found in ornamental goldfish populations? To address to the question, we conducted molecular developmental studies of *szl* gene homologues in goldfish.

In a previous molecular study, we isolated a single *szl* homologue from goldfish (Abe et al., 2014), suggesting that the genome may only contain one *szl* gene. Thus, we assumed that the loss of function of the *szl* gene could not be directly compensated and may then be more lethal than a mutation in one of the two *chd* gene paralogues. Consequently, such a mutant could not become genetically fixed in the population. Moreover, even if the goldfish genome contains two *szl* gene paralogues, it is possible that the genes are functionally redundant, and as such, the depletion of one would not produce the twin‐tail morphology (Sémon & Wolfe, 2007). In the common carp, it was reported that the *chd* gene paralogues are expressed at early embryonic stages in a completely overlapping manner. Thus, the depletion of a single *chd* gene paralogue did not produce any notable phenotypes at late larval stages (Abe et al., 2016; Sémon & Wolfe, 2007). In this report, we examine the presence/absence of *szl* gene paralogues and whether the depletion of the *szl* gene can cause the twin‐tail morphology in goldfish. Based on the results of these experiments, we then consider the rarity of the twin‐tail mutation from the aspect of evolutionary developmental biology.

## 2 MATERIALS AND METHODS

### 2.1 Goldfish strains

Goldfish were purchased from an aquarium fish agency and breeder in Taiwan. To avoid confusion from complicated nomenclature systems (Matsui, Kumagai, & Betts, 1981; Smartt, 2001), individual goldfish with a slender body and a single caudal fin were collectively designated as the single‐tail common goldfish. The single‐tail common‐goldfish individuals were genotyped at the *chdA* locus, and those with allelic combinations were maintained separately. This genotyping method was based on that of a previous report (Abe et al., 2014).

### 2.2 Fish embryos and juveniles

Artificial fertilization was performed using dry methods. Sperm was extracted from individual males and preserved in Modified Kurokura's extender 2 solution (Magyary, Urbanyi, & Horvath, 1996). Activity of the extracted sperm was examined under an upright clinical microscope (BX46, Olympus, Japan) before artificial fertilization. Eggs were squeezed from mature females onto Teflon‐coated dishes. The isolated eggs and sperm were then gently mixed on Teflon‐coated dishes, after which they were transferred to 9‐cm Petri dishes containing tap water (23–24°C). Petri dishes with approximately 50 to 100 fishes were incubated at 24°C until observation and harvest. Embryonic and juvenile stages were determined prior to harvest, according to a goldfish‐staging table (Li, Chang, Liu, Abe, & Ota, 2015; Tsai, Chang, Liu, Abe, & Ota, 2013). All animal experiments were carried out in accordance with protocols that were approved by the Institutional Animal Care and Use Committee at Academia Sinica.

### 2.3 Molecular cloning, sequencing, and phylogenetic analysis

Total RNA was extracted from gastrula‐segmentation stage embryos using TRIzol Reagent (Ambion, USA). PCR primers were designed against regions with conserved amino acid, RNA‐seq, and genome‐sequence data (Abe et al., 2014; Xu et al., 2014; Supporting Information Table S1). After PCR amplification with the primers, products were purified and then ligated into a vector using the TOPO TA Cloning Kit Dual Promoter (Invitrogen, USA), T&A Cloning Vector Kit (Yeastern Biotech, Taiwan), or pGEM‐T Easy Vector system (Promega, USA). The resulting plasmids were used to transform *Escherichia coli* DH5α. Sequenced cDNA fragments were used as backbones for the GeneRacer kit (Invitrogen, USA) to obtain complete sequences by PCR. The isolated sequences were identified by generating multiple amino acid alignments for goldfish with the program CLUSTALW, common carp, and zebrafish. The phylogenetic relationships of goldfish *szl* genes were investigated by reconstructing maximum likelihood trees using MEGA6 software.

### 2.4 Morpholino injection

Antisense morpholino oligonucleotides (MO; Gene Tools, USA) were suspended in water as 1 mM stock solution and diluted with 0.2 M KCl to the appropriate concentration prior to use. Phenol Red (Sigma, USA) was added as an indicator at a final concentration of 0.05%. A microinjector (Eppendorf Femtojet; Eppendorf, Germany) was used to inject 4 nl of MO solution into the yolk of fertilized eggs at the 1 to 2 cell stage. The injected embryos were incubated at 24°C, and morpholino‐injected specimens were phenotyped at the late embryonic stage. Embryos at 2 to 3 days post‐fertilization were categorized based on morphology. All morpholino‐injected specimens were observed under a stereomicroscope (SZX16 and SZ16; Olympus, Japan). To minimize the risk of misinterpreting off‐target effects as being due to MO‐induced knockdown of *szl*, we compared the morphant phenotypes to those of previously reported *ogon/sizzled* and other DV patterning–related gene mutant zebrafish (Hammerschimidt et al., 1996; Kishimoto, Lee, Zon, Hammerschmidt, & Schulte‐Merker, 1997; Muraoka et al., 2006; van Eeden et al., 1996; Yabe et al., 2003). To avoid possible bias from different handling times, the order of MO injections was varied.

### 2.5 In situ hybridization

Digoxigenin‐labeled anti‐sense RNA probes were produced using PCR product templates and the T7 RNA polymerase Riboprobe Combination System (Promega, USA), according to the manufacturer's instructions. The probes were purified using mini Quick Spin RNA Columns (Roche, Switzerland). Primer sets used for the PCR amplification of *szl* fragments are given in a previous report (Abe et al., 2014). PCR product corresponding to a region that includes the relevant 5′ or 3′ untranslated regions (UTRs) were used to generate probes (Supporting Information Table S1).

Whole‐mount in situ hybridization was performed as previously described (Schulte‐Merker et al., 1992), with minor modifications. Fish embryos were fixed with 4% paraformaldehyde in PBS overnight. Embryos were fixed and then dechorionated using fine forceps. After fixation and dechorionation, embryos were dehydrated with methanol. Dehydrated embryos were rehydrated with PBT and fixed with 4% paraformaldehyde in PBS. Embryos were subsequently treated with Proteinase K for 20 min, and then refixed. Pre‐hybridization and hybridization were performed at 65°C for a period that ranged between 1 hr and overnight. The samples were washed sequentially two times with 50% formamide/2 × saline sodium citrate + Tween 20 (SSCT) at 65°C for 30 min each; 2 × SSCT at 65°C for 15 min; and two final washes with 0.2 × SSCT at 65°C for 30 min. The samples were then incubated in blocking solution (10% heat‐inactivated goat serum [Roche, Switzerland], 0.1% Tween‐20 in PBS) for 1 hr, before being incubated with a 1:4,000–8,000 dilution of anti‐digoxigenin‐AP Fab fragments (Roche, Switzerland) at room temperature for 4 hr, or at 4°C overnight. Samples were washed four times with blocking solution at room temperature for 25 min each. Signals were detected using BCIP/NBT Color Development Substrate (Promega, USA). The reaction was stopped by washing samples with 20% MeOH in PBS. To ensure accurate comparison of gene expression levels, the embryos in a single experiment were treated at the same times with identical conditions.

### 2.6 Alizarin red staining

Goldfish juveniles were anesthetized with MS222 (Sigma, USA), and then fixed with 4% paraformaldehyde in PBS. Following fixation, fish were washed in 70% ethanol, stained with alizarin red solution (0.02% alizarin red in 95% ethanol to reduce background [Li et al., 2015]), and finally cleared using Sca*l*eA2 (Hama et al., 2011). The cleared samples were placed on a 0.5% agarose plate and photographed from left lateral and ventral views.

## 3 RESULTS

### 3.1 Identification of *szlA* and *szlB* paralogues

**Figure 1:**
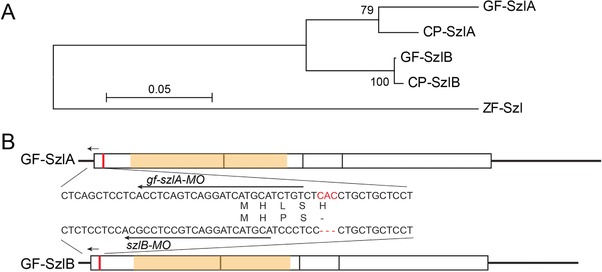
Comparison of molecular sequences of *szl*‐gene paralogues Note: a. Phylogenetic relationship between goldfish *szlA* and *szlB* genes. The phylogenetic tree was reconstructed based on the amino acid sequences using the Maximum Likelihood method. There were a total of 284 positions in the final dataset. DDBJ/EMBL/GenBank accession numbers of goldfish *szlA* and *szlB*, carp *szlA* and *szlB*, and zebrafish *szl* sequences are AB874477, LC341592, LC348963, LC348964, and NM_181663.1, respectively. The prefixes, “GF‐,” “CP‐,” and “ZF‐,” at the operationally taxonomic units indicate goldfish‐, carp‐, and zebrafish‐derived genes, respectively. b. Schematic diagram of goldfish *szlA* and *szlB* mRNA sequences. White boxes indicate exons, orange boxes indicate cysteine‐rich domains, and red and black lines indicate indel difference locus and non‐coding regions, respectively. Arrows indicate the morpholino‐targeting sites for *gf‐szlA‐MO* and *szlB‐MO*. [Color figure can be viewed at http://wileyonlinelibrary.com]

We succeeded in isolating two different *szl* gene‐related sequences, which were designated as *szlA* and *szlB*. The nucleotide sequence of *szlA* is identical with the previously isolated goldfish *szl* gene (DDBJ/EMBL/GenBank accession number, AB874477; Abe et al., 2014). On the other hand, *szlB* (DDBJ/EMBL/GenBank accession number, LC341592) is slightly different from the previously isolated goldfish *szl* gene, with more than 90% similarity between the nucleotide sequences, suggesting that this gene is a paralogue of the *szlA* gene (Abe et al., 2014). To identify when the *szlA* and *szlB* genes arose during evolution, we isolated closely related sequences from common carp and reconstructed the phylogenetic tree of *szl* genes (Figure 1a). In the phylogenetic tree, the goldfish and common carp *szl* genes formed two clusters (*szlA* and *szlB* clusters), with high supportive value, suggesting that the *szl* genes were duplicated in a common ancestor of goldfish and common carp. Moreover, we found a single codon deletion at the fifth codon site in the *szlB* gene (the red letters in Figure 1b), and large differences between the 5′‐ and 3′‐UTR sequences (Figure 1b). These sequence characteristics allowed us to distinguish between the *szlA* and *szlB* genes, as well as providing evidence of their paralogous relationship (Figure 1a). The differences also allowed us to design specific probes for the analysis of gene expression patterns. More importantly, from these results, we can reject the hypothesis that the absence of the *szl*‐type twin‐tail goldfish is due to a lack of *szl* paralogues.

### 3.2 Gene expression patterns of *szlA* and *szlB*

**Figure 2:**
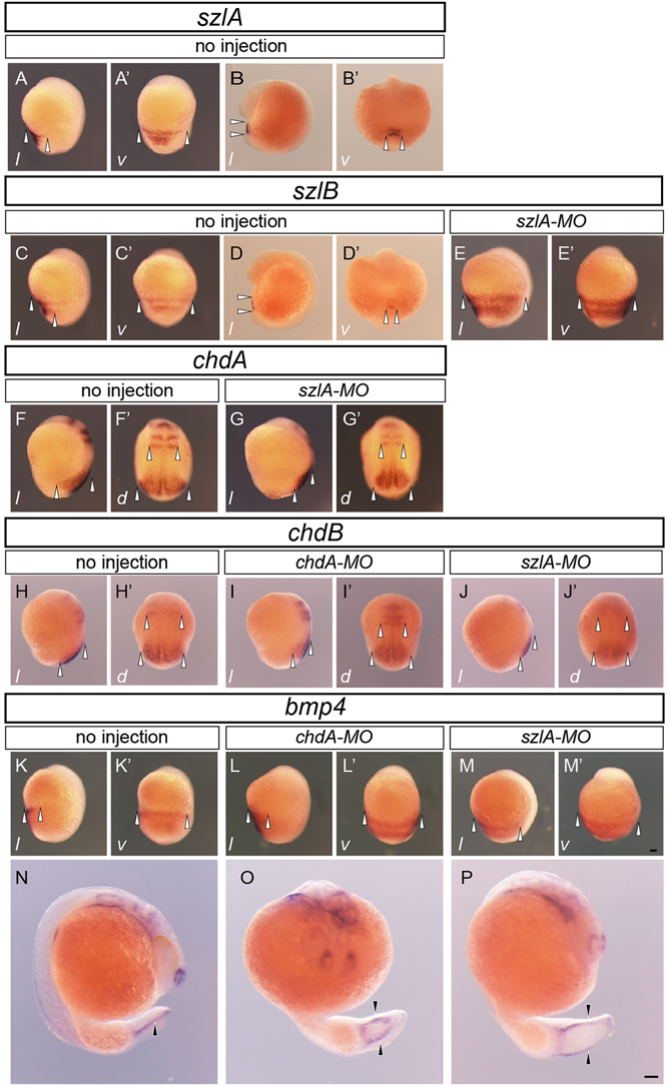
Analyses of gene expression patterns Note: Expression patterns of *szlA*, *szlB*, *chdA*, *chdB*, and *bmp4* are shown in the indicated rows. Bud (a, a’, c, c’, e, e’, f–m’), early segmentation (b, b’, d, d’), and late segmentation stage (n–p) embryos were analyzed for gene expression. Panels e, e’, g, g’, j, j’, and p show *gf‐szlA‐MO*‐injected embryos. Panels i, i’, l, l’, and o show *gf‐chdA‐MO*‐injected embryos. The italicized letters at the left bottom corner of panels from a to m’ represent the orientation of embryos; “*l*,” “*d*,” and “*v*” indicate lateral, dorsal, and ventral views. Panels n, o, and p were photographed from lateral, ventral, and oblique lateral side, respectively. All these panels show ventral views of caudal region. White arrowheads indicate the ranges of gene expression in the early‐stage embryos (a–m’). DDBJ/EMBL/GemBank accession numbers of *chdA*, *chdB*, and *bmp4* are AB874473, AB874475, and AB874475, respectively. The black arrowheads indicate the caudal fin fold primordia. Panels a–m’ and n–o are of the same magnification. Scale bars = 100 μm (m’, p). [Color figure can be viewed at http://wileyonlinelibrary.com]

To answer the question of whether *szlA* and *szlB* have different developmental roles, we first examined the gene expression patterns in single‐tail common‐goldfish embryos (Figure 2a–d′). Both *szlA* and *szlB* were expressed in the posterior ventral region at the bud stage and the segmentation stages, which is consistent with our previous results (Abe et al., 2014; Figure 2a–d′). However, the signal for the *szlA* gene was slightly stronger than that of *szlB* in the ventral region of segmentation‐stage embryos (Figure 2b,b’,d,d’). This difference in expression intensity and/or duration suggests that the *szl* genes may be involved in somewhat different developmental processes.

### 3.3 Comparison of phenotypes between *chordin* and *szl* deficiencies in goldfish

To further investigate how goldfish embryos develop when depleted of *szl*‐gene expression, we designed MOs that differentially target the two paralogues (Figure 1b). The differences between the 5′‐UTR sequences allowed us to target the paralogues using two MO reagents; one is *gf‐szlA‐MO*, which specifically blocks translation of goldfish *szlA* mRNA, and the other is *szlB‐MO*, which may target both *szlA* and *szlB* but shows higher affinity to the *szlB* sequence (Figure 1b). To test the specificity of the *gf‐szlA‐MO*, we injected the MO in embryos of the single‐fin common goldfish and analyzed the gene expression pattern of *szlB* at the bud stage (Figure 2e,e’). Expression of the *szlB* gene was stronger in the *gf‐szlA‐MO*‐injected embryo than in control embryos (Figure 2c,c’,e,e’), suggesting that *gf‐szlA‐MO* only depleted the *szlA* gene product without depletion of *szlB* mRNA.

The increased expression of the *szlB* gene may be due to a negative feedback loop that was previously identified (Muraoka et al., 2006; Yabe et al., 2003). In fact, this result is consistent with the reduced expression patterns of *chdA* gene when *szlA* was depleted (Figure 2f,f’,g,g’). Moreover, to compare the differences of *chdA‐* and *szlA*‐depleted embryos, we also observed the expression patterns of *chdB* gene. In *chdA*‐depleted embryos, *chdB* expression was reduced only in lateral regions, whereas in *szlA*‐depleted embryos there was an overall reduction of expression intensity and area for the *chdB* gene (Figure 2h–j’). Furthermore, we also observed expression pattern of the ventral‐marker gene (*bmp4*; Figure 2k–p). In *gf‐chdA‐MO*‐injected embryos, both enhanced intensity and lateral expansion of expression *bmp4* gene expression were observed (Figure 2l,l’). On the other hand, only laterally expanded expression patterns were found in *gf‐szlA‐MO*‐injected embryos, while enhancement of *bmp4* expression intensity was not observed (Figure 2m,m’). The differences in expression intensity between the two groups of early‐stage embryos may be explained by previous studies in zebrafish, which showed that *chd* directly antagonizes *bmp4*, but *szlA* does not (Muraoka et al., 2006; Yabe et al., 2003). In contrast to the bud‐stage embryos, late‐segmentation‐stage embryos with *chdA* or *szlA* depletion exhibited the same phenotype (Figure 2n–p). Bifurcated *bmp4* signals were observed in both *chdA‐* and *szlA*‐morphant embryos at segmentation stage, suggesting that depletion of either *chdA* or *szlA* can similarly reproduce the bifurcated caudal fin fold (Figure 2n–p).

**Figure 3:**
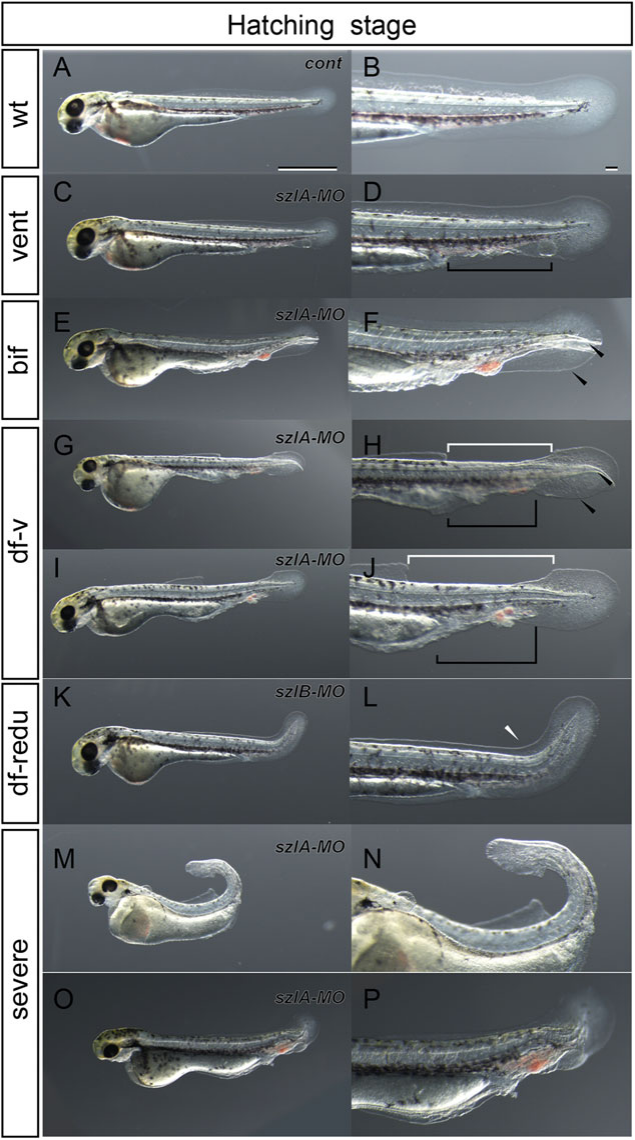
Phenotypic variation of *szl*‐depleted goldfish at hatching stage Note: Left panels (a, c, e, g, i, k, m, and o) show wild‐type, weakly ventralized, bifurcated caudal fin fold, dorsal fin fold less bifurcated caudal fin, dorsal fin fold less ventralized, dorsal fin fold reduced, curled tail, and highly ventralized embryos. Right panels (b, d, f, h, j, l, n, and p) show magnifications of the caudal regions of the respective left panels (a, c, e, g, i, k, m, and o). Italicized descriptions, *cont*, *szlA‐MO*, *szlB‐MO*, and *chdA‐MO* in panels a, c, e, g, i, k, m, and o indicate control, *szlA‐MO*‐injected, *szlB‐MO*‐injected, and *chdA‐MO*‐injected individuals, respectively. Black arrowheads, white arrowheads, black arrows, black brackets, and white brackets indicate bifurcated fin fold, reduced dorsal fin fold, bifurcated fin, malformed region on the ventral side, and malformed region on the dorsal side, respectively. Scale bars = 1 mm (a), 0.1 mm (b). Left panels (a, c, e, g, i, k, m, and o) and right panels (b, d, f, h, j, l, n, and p) are shown at the same magnification. [Color figure can be viewed at http://wileyonlinelibrary.com]

To further examine the effect of *szl* depletion at later embryonic stages, the *gf‐szlA‐MO*, *szlB‐MO‐* and *chdA‐MO*‐injected embryos were raised until pre‐hatching stages (Figure 3a–p). Both *szlA* and *szlB* morphants exhibited typical ventralized phenotypes (weakly‐ventralized and bifurcated‐caudal‐fin phenotypes) at the hatching stage (Supporting Information Figure S1). Thus, the *szl*‐morphant phenotypes mimicked *dino/chordin* and *ogon/sizzled* zebrafish, as well as *chdA*‐morphant and twin‐tail goldfish (Abe et al., 2014; van Eeden et al., 1996; Yabe et al., 2003; Figure 3c–f).

In addition to the shared ventralized phenotypes, we observed *szlA‐* and *szlB*‐morphant‐specific phenotypes (Figure 3g–l). For example, some larvae with weakly ventralized (vent) or bifurcated fin folds (bif) also lacked a part of the dorsal fin fold (Figure 3g–j). Moreover, several *szlB* morphants simply showed a reduction of the dorsal fin fold (Figure 3k,l). In more extreme cases, the entire body of the larva was affected by the depletion of *szl*, as illustrated in Figure 3m,n, by an individual that exhibited a curled caudal region with malformations of the heart and head regions. These phenotypes in the *szl*‐morphant goldfish were morphologically quite similar to the typical dorsalized phenotypes of zebrafish mutants (called the C2 and C3 phenotypes), suggesting that the goldfish *szl* gene morphants exhibit at least some features of over‐dorsalized phenotypes (Figure 3m,n; Kishimoto et al., 1997). The differences between the *szl* and *chd* morphants might reflect functional differences between *chd* and *szl* genes that have been described in early zebrafish embryos and were suggested by our *bmp4* expression analyses (Muraoka et al., 2006; Yabe et al., 2003; Figures 2h–j’ and 3m,n).

Based on previously reported phenotypes of zebrafish mutants and twin‐tail goldfish, we grouped the morphants into four phenotypic categories, including wild‐type, vent, bif, and dorsal fin mutation (dorsal; Figure 3a–p; Abe et al., 2014; Hammerschmidt et al., 1996; Kishimoto et al., 1997; van Eeden et al., 1996). The dorsal phenotype was further partitioned into three subcategories: dorsal fin reduced with ventralized, dorsal fin reduced with bifurcated fin fold, and dorsal fin reduced. Using these criteria, we further examined the functional effects of *szlA* and *szlB* genes at the hatching stage. The penetrance of these phenotypes was dependent on the concentration and combination of morpholino reagents and showed the same trends in three independent experiments (Supporting Information Figure S1). Across three independent clutches, a total of six types of comparable microinjections were performed (Supporting Information Figure S1). No matter which concentration of injected MO was used, *gf‐szlA‐MO*‐injected groups always exhibited a higher proportion of non‐wild‐type fish than *szlB‐MO*‐injected groups (Supporting Information Figure S1). Even though *szlB‐MO* is expected to have some non‐specific affinity for *szlA* mRNA, this milder morphant phenotype for *szlB‐MO* corresponds to narrower expression patterns for *szlB* in comparison with the *szlA* (Figure 2a–d’).

**Figure 4:**
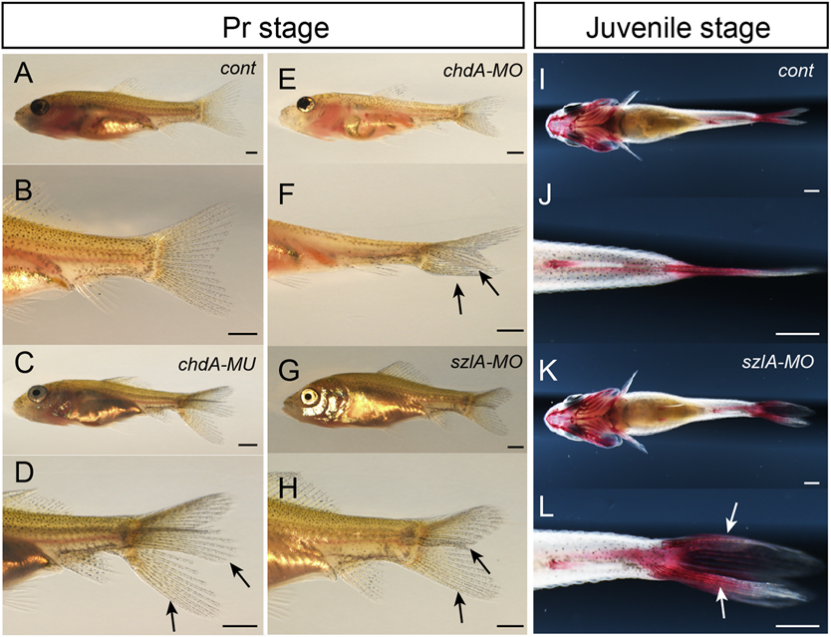
Bifurcated caudal fins of *szl*‐depleted goldfish Note: a–h. Lateral oblique views of Pr‐stage goldfish larvae. i–l. Ventral views of alizarin red–stained juveniles. Panels b, d, f, h, j, and l show magnified views of a, c, e, g, i, and k, respectively. Italicized descriptions, *cont*, *szlA‐MO*, *szlB‐MO*, *chdA‐MO*, and *chdA‐MO* in panels a, c, e, g, i, and k indicate control, *szlA‐MO*‐injected, *szlB‐MO*‐injected, *chdA^E127X/E127X^* mutant, and *chdA‐MO*‐injected individuals, respectively. Black and white arrows indicate bifurcated caudal fin. Scale bars = 1 mm. [Color figure can be viewed at http://wileyonlinelibrary.com]

To investigate whether any morphant exhibited bifurcated caudal skeletons, injected fish were observed at late larval stages, specifically the pelvic fin ray stages wherein fin rays and calcified axial skeletal tissues are already formed (Li et al., 2015; Figure 4a–h). Our observations suggest that the *szlA* morphant exhibits somewhat similar caudal fin morphology to *chdA* mutants and *chdA* morphants at pelvic fin ray stages. Moreover, the *szlA*‐morphant larvae could grow to juvenile stages and clearly exhibited a bifurcated caudal fin (Figure 4i–l). Fourteen individuals were maintained in our aquarium facility, of which eight survived to 12 months. Five of these eight individuals exhibited bifurcated caudal fin rays. Thus, depletion of *szl*‐gene expression in goldfish could reproduce the bifurcated caudal fin morphology found in twin‐tail goldfish and *dino* or *ogon* zebrafish (Muraoka et al., 2006; Yabe et al., 2003; Figure 4a–l).

## 4 DISCUSSION

Our study reveals that the *szl* gene was duplicated in the common ancestor of goldfish and common carp, consistent with previous reports detailing whole draft genome sequencing of common carp and our molecular sequence analysis of *chd* paralogues (Figure 1; Abe et al., 2014; Xu et al., 2014). Moreover, differentiated expression patterns of *szlA* and *szlB* paralogues were observed in segmentation‐stage embryos (Figure 2b,b′,d,d′), similar to the expression patterns of *chd* paralogues (Abe et al., 2014). The coding sequences of these paralogues are closely related and the *szl* gene is known to be transcriptionally regulated by *bmp*‐downstream effectors in zebrafish embryos (Muraoka et al., 2006; Yabe et al., 2003). Therefore, it is reasonable to assume that the observed differentiated expression patterns are based on differences in *cis*‐regulatory regions.

The successful production of a twin‐tailed phenotype with morpholino‐induced knockdown of *szlA* allows us to conclude that the observed expression pattern differences between *szl* paralogues are derived from subfunctionalization, rather than partial redundancy, incomplete non‐functionalization, or neo‐functionalization (Figures 2, 3, 4). In fact, the injection of MOs that target either of the *szl* paralogues in single‐tail common‐goldfish embryos was sufficient to reproduce the bifurcated caudal fin and its primodia (Figures 2 and 3e,f; Supporting Information Figure S1). Therefore, the *szl* paralogues are not functionally redundant and cannot rescue each other, unlike other paralogous gene sets that exhibit partial redundancy, incomplete non‐functionalization, or neo‐functionalization (Figure 3; Abe et al., 2016; Force et al., 1999; Innan & Kondrashov, 2010; Rastogi & Liberles, 2005; Sémon & Wolfe, 2007). Our results suggest that the absence of *szl*‐type twin‐tail goldfish in domesticated populations is not due to buffering mechanisms between *szl* paralogues. Therefore, molecular developmental studies cannot directly answer the question of why only *chordin*‐type twin‐tail goldfish can be observed.

Several different types of stochastic events may potentially explain the lack of observed *szl*‐type twin‐tailed goldfish. For example, there is a possibility that the *szl*‐type twin‐tail goldfish actually does exists in domesticated and/or natural populations, but it was somehow absent from investigated populations. Moreover, since the twin‐tail phenotype would be derived from a gene loss event at an *szl* locus, the rate of the gene loss and the timing of allotetraploidization are important factors to evaluate. Recent *Xenopus* genome studies have indicated that more than 56% of all genes were retained as two homologous copies after speciation 34 million years ago and allotetraploidiztion 17 million years ago (Session et al., 2016). This high retention rate suggests that very little gene loss may have occurred in the goldfish lineage (especially with reference to *szl* paralogues), due to the short divergence time after allotetraploidization (8.2 million years ago; Abe et al., 2014). To distinguish whether the absence of *szl*‐type twin‐tail goldfish is simply due to a stochastic event, the frequency of gene loss after allotetraploidization may be estimated by comparing whole genome sequences between goldfish and its relatives in further studies.

In addition to potential stochastic processes, we can consider a hypothetical case where a nonsense mutation occurs in the *szlA* gene during ornamental goldfish domestication. Because we observed quite similar bifurcated caudal fin morphology in *chdA* mutants and *szlA* morphants at the late larval stage (Figure 4a–l), the *szl*‐type twin‐tail goldfish would be expected to exhibit sufficiently attractive bifurcated caudal fin morphology for breeders. Thus, even though the expressivity of the bifurcated caudal fin morphology was probably not high in ancestral stocks, breeders may have attempted to genetically fix the morphological features that they most preferred. However, it is uncertain whether the *szlA*‐type twin‐tail goldfish would be as physically viable as the *chdA*‐type twin‐tail goldfish, based on the different embryonic phenotypes that we observed (Figures 3 and 4). As such, the *chdA* morphant tends to show mutant phenotypes only on the ventral side, while the *szlA* morphant exhibits mutant phenotypes on both dorsal and ventral sides of hatching stage embryos (Figure 3c–j; Supporting Information Figure S1). These malformations of both dorsal and ventral sides in the *szlA*‐morphant embryos seem to be caused by the destabilization of DV‐patterning mechanisms, rather than a simple ventralization of the caudal region. In fact, the *szlA*‐morphant phenotypes at hatching stage may be derived from altered expression of the *chdB* gene at the bud stage (Figures 2h–j’ and 3c–j). Unlike the *chdA* morphant, the *szlA* morphant exhibited substantially attenuated *chdB* expression, suggesting that the reduction of dorsal tissues resulted from defects in the bud‐stage embryo. Consequently, the viability of an *szlA‐*mutant goldfish may be lower than that of the *chdA* mutant. This result is contrary to our expectation, as it does not reflect the higher survival rate of *ogon/sizzled* zebrafish compared to *dino/chordin* zebrafish (Fisher & Halpern, 1999; van Eeden et al., 1996). Thus, during domestic breeding, the more viable *chd*‐type twin‐tail goldfish would be preferred, intensively selected, and consequently genetically fixed in the ornamental goldfish population. Notably, during the time of twin‐tail selection, sophisticated aquarium systems were unavailable to early breeders, who worked to develop and maintain ornamental goldfish strains in the period from the Song to Ming dynasty, between 1000 and 1600 c.e., approximately (Chen, 1956; Ota & Abe, 2016; Smartt, 2001). On the other hand, *szlB* may also be mutated to produce a goldfish with the twin‐tail phenotype. However, in *szlB‐MO*‐injected goldfish, the twin‐tail phenotype is reproduced with low efficiency, leading us to conclude that the expressivity of the *szlB*‐mutant phenotypes might be lower than that of the *chdA‐* and *szlA‐*mutant phenotypes (Supporting Information Figure S1). Based on this assumption, it would also be expected that the *chdA‐*type twin‐tail goldfish would be preferred over the *szlB* type twin‐tail goldfish for selection. Breeders will generally prefer a parent that stably transmits the twin‐tail morphology to offspring (Matsui et al., 1981; Smartt, 2001).

Based on the results of our *szl*‐knockdown experiments, we conclude that the *szl*‐type twin‐tail goldfish would probably not be artificially selected during domestic breeding over a *chdA*‐type twin‐tail goldfish, even if the *szl* mutation were to occur. Moreover, the non‐preferred phenotypes that we observed in *szl* morphants suggest that the uniqueness of the twin‐tail goldfish is due to the exceedingly uncommon occurrence of a genetic mutation that can cause a large‐scale morphological change in the axial skeleton with high expressivity and high viability. Our results can be generalized to conclude that even though there may be multiple possibilities for different gene mutations to generate the same type of large‐scale molecular developmental and morphological changes, only a few of these possible mutations would be acceptable for genetic fixation in a population due to the selective pressures from breeders and the husbandry environment. In addition to domesticated populations, the present study on goldfish may also provide valuable insights into how large‐scale morphological changes may occur during evolution under natural conditions.

## CONFLICT OF INTEREST

None.

## Supporting information

SUPPORTING INFORMATION
