# Supplementary material for: An alternative evolutionary pathway for the twin‐tail goldfish via szl gene mutation
Source: J Exp Zool B Mol Dev Evol. 2018 Jun 27;330(4):234–41. doi: 10.1002/jez.b.22811 (PMC6033011; doi:10.1002/jez.b.22811)
Supplement: Supplementary file 1 — SUPPORTING INFORMATION [file JEZ-330-234-s001.pdf]

Supporting Materials

Title: An alternative evolutionary pathway for the twin-tail goldfish via *szl* gene mutation

Authors:

Gembu Abe[1, 2, †], Shu-Hua Lee[1, †], Ing-Jia Li[1], and Kinya G. Ota[1*]

Affiliations:

[1]Laboratory of Aquatic Zoology, Marine Research Station, Institute of Cellular and Organismic Biology, Academia Sinica, Yilan, 26242, TAIWAN.

[2]Laboratory of Organ Morphogenesis, Department of Developmental Biology and Neurosciences, Graduate School of Life Sciences, Tohoku University, Aobayama Aoba-ku, Sendai 980-8578, JAPAN.

Total number of supplemental figures: 1

Total number of supplemental tables: 1

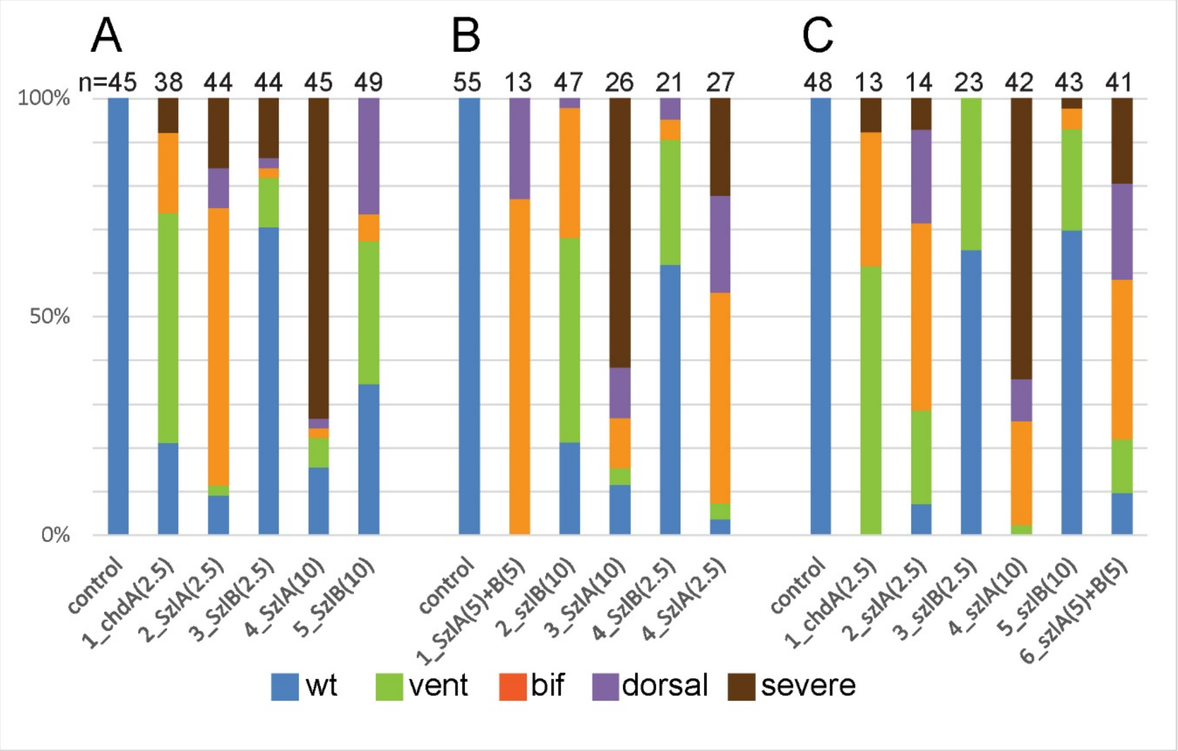

Supplementary Figure S1: Proportions of morphant phenotypes following injection of embryos.

Injected morpholinos are indicated under each bar of graphs showing the first (**A**), second (**B**), and third (**C**) clutches. Prefix numbers indicate the order of injection, and numbers in brackets indicate the amount of injected morpholino (ng). The number of individuals analyzed is indicated above each bar. The abbreviations “wt”, “vent”, “bif”, “dorsal” and “severe” indicate wild-type, weakly ventralized, bifurcated caudal fin, phenotypes showing malformation in dorsal side and severe phenotypes, respectively. Phenotypes of dorsal fin fold less with bifurcated caudal fin, dorsal fin fold less with weakly ventralized, and dorsal fin fold reduced, which are shown in **Fig 3**, are collectively categorized as dorsal phenotype. Curled tail and highly ventralized phenotypes are grouped as severe phenotype.

Supplementary Table S1: Primer list

gene	Primer name*	sequence (5'->3')
szlA	GF-szlA-SP6	CAGTGAATTGATTTAGGTGACACTATAGAAGTGGA GATGCGTCTGCCGAACCTG
	GF-szlA-T7	TAATACGACTCACTATAGGGAGAGCTGTAATCTCT ATGTACAACAGTCGG
szlB	Carp-GF-szlB-in situ-SP6	CAGTGAATTGATTTAGGTGACACTATAGAAGTGCTC TCCTCCACGCCTCCGTCAGG
	Carp-GF-szlB-in situ-T7	AATACGACTCACTATAGGGAGAGGTACAACGGTC AGAGTGCATCTC
	Carp-GF-szlB-3’-F1	ATTCCCTCAGCCTGTGTGCCAA
	Carp-GF-szlB-3’-F2	AGACTTTGCCGTGAAGGT
	Carp-GF-szlB-5’-R1	ACAACACGGGAGCGCAGCTCTCCT
	Carp-szlB-5’-T7-R2	TTCACGGCCACACACACGCTCCGG
	Carp-GF-szlB-full length-F2	CTCTCCTCCACGCCTCCGTCAGG
	Carp-GF-szlB-full length-R2	ACAGTAGAAACATTTATTATTTACAGCAC

* The prefix "GF", "Carp" and "Carp-GF" indicate the goldfish specific-, carp specific- and commonly available primers, respectively.
